# Natural Disasters, Psychosocial Distress, Psychological Flexibility, and Satisfaction with Life

**DOI:** 10.3390/bs15070848

**Published:** 2025-06-24

**Authors:** Rodger K. Bufford, Javeen Skoubo, Kenneth Logan, Aundrea Paxton

**Affiliations:** Graduate School of Clinical Psychology, George Fox University, Newberg, OR 97132, USA; javeenbeard@gmail.com (J.S.); klogan@georgefox.edu (K.L.); dr.aundreapaxton@gmail.com (A.P.)

**Keywords:** natural and human (Adverse Childhood Experiences) disaster, maltreatment, trauma, simple and complex PTSD, social support, satisfaction with life, psychological flexibility

## Abstract

Although common, natural disasters (NDs) remain little studied, and their aggregate psychological impact is unknown. No aggregate measure of ND exposure could be located. This study reports the development and preliminary validation of the Natural Disaster Scale (NDS). In a sample of 131 US adults, disaster exposure was measured for NDs, for childhood and adult human disasters, for psychological flexibility, and for social support. Criteria included general distress, simple and complex PTSD, and life satisfaction. The NDS showed good internal consistency. Eleven of thirteen items loaded on a single factor. After controlling demographic factors, social support, and psychological flexibility, the NDS predicted general distress, simple PTSD, and satisfaction with life. In comparison, adult human adversity/disaster predicted general distress and simple PTSD, while childhood human adversity/disaster predicted complex PTSD and satisfaction with life. Similarly sized effects were found for human and natural disasters, except that only childhood adversity predicted complex PTSD. Demographic factors were insignificant except that age predicted life satisfaction. Because NDs may lead to distress, simple PTSD, and diminished life satisfaction, appraising lifelong exposure to NDs may be important for treatment. Those exposed to NDs may benefit from trauma-informed care. Fostering psychological flexibility, as proposed in the ACT therapy, is suggested as an important treatment focus for addressing the effects of human and natural disasters.

## 1. Impact of Natural Disasters on Psychosocial Distress and Life Satisfaction

It was a dark and stormy day. The wind blew and rain fell. As the hours passed, the day grew darker. The wind increased in intensity, and the rain fell more heavily. Suddenly, the front door flew open, banging against the wall. Wind and rain blasted through the open door. Soon, the door blew open again. And again. After four or five unsuccessful efforts to keep the door closed, the couch was placed against the door, and the door remained shut. But still, the storm’s intensity increased. Hurricane Hazel came ashore on 15 October 1954, with winds up to 150 miles per hour. We survived. But how did the storm affect us? How do other natural disasters affect us?

Hurricane Hazel is but one storm and one example of the many forms of natural disasters (NDs). Several studies have examined the psychological aftermath of individual events that involve NDs. But what if they come repeatedly? Avalanche, earthquake, fire, flood, hurricane, tornado, typhoon—NDs can take many forms and can affect an individual repeatedly.

In discussing adverse childhood experiences and exposure to potentially harmful and distressing NDs, we are faced with a linguistic dilemma. The word *trauma* originally referred to bodily harm or injury following events such as a fall, car crash, or engagement in combat. Extending trauma to include psychological injuries and other sequelae of disasters seems logical. But *trauma* is now widely used to refer to events such as bullying that may precipitate such harm as well. This can be confusing. Bromet et al. (2017) introduced the language of human disaster (HD) and natural disaster. For clarity, in the following material, *disaster* will be used to identify precipitating events, whether by human agents, such as physical or sexual abuse, or through natural processes, such as earthquakes and tornadoes. In the following material, trauma language will refer to human injury following such disasters.

The traumatic effects of individual NDs are well explored. For example, Clemens et al. (2013) examined the effect of the 2010–2011 flooding and cyclones on well-being among residents in Queensland, Australia. Self-Brown et al. (2013) reported traumatic outcomes of Hurricane Katrina. Hamama-Raz et al. (2017) reported on trauma in the aftermath of the November 2013 Philippine Typhoon, Haiyan. Mesidor and Sly (2019) reported on trauma after the 2010 earthquake in Haiti. Sun et al. (2019) reported on the impact of earthquakes. Similarly, Kimura (2022) reported on the human toll of the 2011 Great East Japan Earthquake, and Narayan et al. (2021) reported that recovery was common for children following NDs or exposure to human disasters, but NDs and human disasters may also have lasting effects. For example, Adebäck et al. (2022) found young adults exposed to a 2004 tsunami in Southeast Asia still experienced external or internal reminders of the tsunami nine years later. Participants could generally manage these effects using various coping strategies. But the effects lingered. Kira et al. (2008) reported that such effects are cumulative.

The impact of NDs also varies among individuals. Witt et al. (2024) reported that while signs of resilience and recovery were most common in the studies they examined, “female gender, a higher trauma exposure, more life events, less social support, and negative coping emerged as risk factors” (p. 1) for chronic distress. Hamama-Raz et al. (2017) reported that positive reframing predicted higher scores, while self-blame predicted lower scores on subjective well-being. Similarly, Fayaz (2023) reported posttraumatic growth among children in the aftermath of “natural disasters” (p. 305). But Fayaz contended that NDs also have adverse impacts on families and communities that commonly result in subsequent human-induced disasters that exacerbate the impact of NDs on personal functioning.

Kira used the word trauma to describe what we here call disaster (Kira, 2001; Kira et al., 2008). Trauma has complex roots. Kira’s (2001) taxonomy of trauma distinguished “nature-made or man-made trauma” (p. 84).

Kira distinguished between indirect and direct disasters; indirect disasters are passed on by others or by one’s culture. He proposed that “*direct traumas*” can be classified into simple and complex trauma, and that complex trauma takes two forms—a complex array of traumatic elements in a single event or an ongoing series of events. For Kira, Type I involves a single event, while Type II involves a series of repeated and connected events; Type III involves “a cascade of traumatic events” (p. 82) that may be direct or indirect and may affect one or more areas of personal functioning—attachment, autonomy/identity, interdependence, achievement/self-actualization, and survival.

Unfortunately, Kira primarily addressed human disasters. Kira’s twenty-item Cumulative Trauma Scale-Revised was developed to measure disaster exposure or “cumulative trauma dose” (Kira et al., 2008, p. 62). But only a single item assessed “natural trauma.” Beard (2023) reported that omitting this item from the Cumulative Trauma Scale-Revised had a negligible effect on scores; the Cumulative Trauma Scale-Revised without this item correlated 0.966 with the full twenty-item scale. Thus, the psychological sequelae of natural disasters remain little explored except for the impact of singular events. Methods to assess exposure to NDs are generally simplistic and debatable (Harville et al., 2015).

Harville et al. (2015) developed the Brief Trauma Questionnaire, a “detailed inventory” that used an interview to explore exposure to hurricanes and flooding. Though limited to these two forms of natural disaster, they concluded that a single question was only about two-thirds as effective as the more detailed questionnaire. They stated, “A single broad question is unlikely to be useful for assessing the degree of exposure to disaster … and varies in utility depending on the mental health outcome of interest …” (p. 1). An important limitation of current ND research is that it is largely limited to single NDs and mostly poses a single question. Furthermore, self-reported exposure appears to be a more reliable indicator of an ND’s impact than merely residing in an affected community—the effects of NDs are not uniformly spread among community members, and individual differences in response to NDs also matter.

Human disasters are extensively studied. Human disaster during childhood is assessed with the Adverse Childhood Experiences (ACE) scale (Felitti et al., 1998). It includes ten items and has been used extensively. Numerous variants of the scale have also been derived (e.g., the ACE-IQ used by the World Health Organization; Anda et al., 2010). ACEs’ impact on well-being have been examined and extensively documented (e.g., Widom et al., 2007). The Cumulative Trauma Scale-Revised (Kira et al., 2008) assesses adult human disasters.

A search for a measure of natural disaster proved unsuccessful. Thus, there remains a need for a scale that enables exploration of complex patterns of ND exposure that parallels the adverse childhood experiences literature for human disasters.

In a recent meta-analysis of the qualitative literature on natural disaster outcomes in children and adolescents, Fayaz (2023) identified twenty-one articles that met criteria for inclusion in the study. One suggestion of Fayaz was that future studies should use moderation analysis in exploring the aftermath of disaster exposure, as the relationships among disaster, PTSD, post-traumatic growth, and other trauma variables are complex and may be influenced by disaster type, age of the affected individual, and other individual factors.

Social support may ameliorate HD- and ND-related distress (e.g., House, 1981; Prakash & Srivastava, 2020; Shakespeare-Finch et al., 2019). Charuvastra and Cloitre (2008) found that perceptions of low social support predicted the development of PTSD; they concluded that disasters of a personal nature had more potential for traumatic effects than those of a non-personal nature, such as a fire or a motor vehicle crash. Also, Kaniasty (2012) found that post-disaster social support fostered psychological well-being, and Woodward et al. (2015) found that social support was a protective factor following disaster exposure. More recently, Kimura (2022) found that “mutual assistance,” likely a form of social support, moderated distress following the 2011 Great East Japan earthquake. But an Israeli study (Shalev & Freedman, 2005; in Charuvastra & Cloitre, 2008) found no symptomatic differences between survivors of motor vehicle crashes and those of terrorist attacks when appraised one week after the event.

Because they are more likely to disrupt social support or induce moral injury (Litz et al., 2018, 2009; Thomas et al., 2024), HDs may have a greater traumatic impact than NDs (Charuvastra & Cloitre, 2008). Merrell (2013) and Merrell et al. (2013) compared the distress among individuals exposed to human and natural disasters in an international context. Results showed no differences in scores on the Impact of Events Scale-Revised or on the Dissociative Events Scale Taxon (DES-T) items (Waller et al., 1996). However, an analysis of item responses on the Impact of Events Scale-Revised revealed significant differences in scores for five items, indicating specific symptoms reported following NDs differed from those following HDs. These differences were obscured when examining Impact of Events Scale-Revised scores for the total scale. Results for the DES-T were similar: no difference was found for the total score, but significant differences between participants with HD and ND histories were found for four of the DES-T items.

Psychological flexibility is a central goal of ACT therapy. ACT therapy is considered the third wave of cognitive behavioral psychotherapy. ACT combines elements of mindfulness with cognitive behavioral techniques. A central premise of ACT is that we are all exposed to illnesses, griefs, and other disappointments in life. Efforts to avoid experiencing these challenges are common but harmful to biopsychosocial functioning (Dindo et al., 2017). Research in support of ACT shows that those who develop mindfulness and the psychological flexibility to accept (Acceptance) and continue their pursuit of valued life goals (Commitment) function better than those who do not (Dindo et al., 2017). ACT involves six inter-related processes: Acceptance, cognitive defusion (i.e., accepting distressing experiences rather than becoming paralyzed by or denying them), being present, self as context, values, and committed action (Luoma et al., 2017). Psychological flexibility is described as a functional behavior that can facilitate achieving goals consistent with one’s values through Acceptance and adaptation/Commitment. Thus, psychological flexibility may provide a buffer that can reduce the negative impact of human and natural disasters on psychological and biosocial functioning (Gloster et al., 2017).

Tindle and Moustafa (2021) proposed that psychological flexibility could function as a moderator to reduce the experience of anxiety, depression, and other forms of psychological distress following disasters such as COVID-19. Results support this hypothesis. For example, Ong et al. (2021) showed that psychological flexibility moderated the effects of perfectionism on quality of life and psychological symptoms. Dawson and Golijani-Moghaddam (2020) found psychological flexibility predicted better mental health and well-being in the UK during the pandemic. Similarly, Chisari et al. (2022) showed that psychological flexibility reduced depression and pain among chronic pain patients. As is common among cognitive behavioral psychotherapies, ACT therapy techniques are emphasized rather than relationships. However, abundant evidence from common factors research demonstrates that ACT therapists engage in relationship development; it is commonly thought that relationships play a central role in the success of ACT interventions (e.g., Cuijpers et al., 2019). ACT is a transdiagnostic and flexible empirically supported psychotherapy that offers “a promising way to meet the heterogeneous needs and treatment preferences of patients with a broad range of problems, including mental health, medical, behavioral, and co-occurring mental and physical health symptoms” (Dindo et al., 2017, p. 11).

Finally, quality of life is a widely studied marker of well-being (e.g., Dhungana et al., 2021). Thus, it can be expected that human and natural disasters will adversely affect the quality of life.

### Problem Statement

The first purpose of the present study is to explore the development of a scale to measure the aggregated impact of ND exposure over the lifetime of participants. A second purpose was to compare the relative impact of HDs and NDs, considering contradictory evidence regarding their relative impact.

## 2. Method

### 2.1. Participants

A national survey yielded a sample of 131 participants. Missing data were replaced in four instances where one response was missing for the participant. One missing ACE response was replaced (<0.001% of ACE responses); one missing response for the NDS was replaced (<0.001% of NDS responses); finally, for the CTS, two responses were replaced (<0.001%), each with the modal response for that item.

Among participants, 75 (57.3%) were female, and 54 (41.2%) were male; one participant did not respond. Participants were relatively young; almost half were less than 34 years old. Age was gathered by categories of 10 years; 49 (37.7%) participants were less than 25 years old and formed the modal age category, 37 (28.2%) were 25–34 years old, 24 (18.3%) were 34–45, 13 (9.9%) were 45–54, 6 (4.6%) were 55–64, and 1 (0.8%) was 65 or older (median age 25–34).

Among participants 88 (67.2%) self-identified as White;; 9 (6.9%) identified as African American, 16 (12.2%) as Hispanic, 6 (4.6%) as Asian/Asian American, 1 (0.8%) as American Indian or Alaska Native, 7 (5.3%) as racially mixed, and 3 (2.3%) as Other. One participant did not respond to this question.

Participants were more educated than the general U.S. population; 35 (26.7%) reported a high school diploma or GED, 32 (24.6%) reported some college up to an Associate’s degree—the median fell in this category, 39 (29.8%) reported a Bachelor’s degree—the modal category, and 24 (18.5%) reported post Baccalaureate education.

In terms of income, 9 (6.9%) reported incomes of less than $15,000; 17 (13.0%) reported incomes between $15,000 and $29,999; 31 (23.7%) reported incomes from $30,000 to $49,999 and both mode and median were in this category; 26 (19.8%) reported incomes from $50,000 to $74,999; 15 (11.5%) reported incomes from $75,000 to $99,999; 20 (15.3%) reported income from $100,000 to $149,999; and 11 (8.4%) reported incomes greater than $150,000. Two did not respond to this item.

### 2.2. Materials

In addition to providing demographic information that included age, gender identity, ethnic identity, education, and income, participants completed measures of human and natural disaster, moderator variables that included psychological flexibility and perceived social support, and criterion measures of trauma and satisfaction with life.

#### 2.2.1. Predictors

**Adverse Childhood Experiences Scale (ACE).** The ACE is a well-known and widely used measure of potentially harmful events experienced between birth and age eighteen (Felitti et al., 1998). Many variations of the ACE scale have been developed (Kostić et al., 2019). This study used the original ACE developed by Felitti et al. (1998). It has ten items that assess exposure to physical and emotional neglect, physical and emotional abuse, and household dysfunction. *Yes/No* responses indicate whether the individual experienced that type of trauma as a child. Scores can range from 0 to 10, indicating the number of kinds of trauma the individual experienced. Bufford et al. (2017) reported an internal consistency coefficient alpha of 0.77 for the ACE. Alpha was 0.84 in the present study.

**Cumulative Trauma Scale-Revised (CTS-R).** The Cumulative Trauma Scale was developed by Kira (Kira, 2001; Kira et al., 2008) to assess the impact of distressing events such as parental abandonment, physical and sexual abuse, rape, torture, war. There is some overlap in the ACE and Impact of Events Scale-Revised in the domains sampled, but whereas the ACE assesses only events before 18 years of age, the Cumulative Trauma Scale-Revised includes adult experiences of adversity. The original scale was based on Kira’s (2001) taxonomy of complex trauma.

The CTS-R (Kira et al., 2008) is a shortened version of Kira’s lengthy Cumulative Trauma measure. Twenty items assess experiences such as family trauma, collective identity trauma, personal identity trauma, and survival trauma. Merrell et al. (2013) reported that the Cumulative Trauma Scale-Revised includes one item that asks if participants have experienced a natural disaster: “I witnessed or experienced a natural disaster.” The remaining nineteen items assess various forms of human adversity, and the Cumulative Trauma Scale-Revised, with the lone natural disaster item, correlated 0.996 with the full Cumulative Trauma Scale-Revised scale; thus, the Cumulative Trauma Scale-Revised is a human adversity measure. Responses are *YES/NO*, parallel to the binary ACE responses. Scores can range from 0 to 20. Merrell reported a coefficient alpha of 0.80 for the Cumulative Trauma Scale-Revised (Merrell, 2013; Merrell et al., 2013). In the present study, alpha was 0.89.

**Natural Disaster Scale (NDS).** The NDS is a new scale developed for this study and first reported by Beard (2023). Merrell et al. (2013) reported that the Cumulative Trauma Scale-Revised included only a single item that assessed exposure to natural trauma (“I witnessed or experienced a natural disaster”). Thus, an initial list of natural trauma items was developed by the research team. The scale employed in this study consisted of thirteen items. To parallel the ACE, binary *Yes/No* response choices were given. Coefficient alpha in the present study was 0.93. Supplemental Table S1 reports item responses for the NDS items.

#### 2.2.2. Moderators

**Acceptance and Commitment Scale-2.** The Acceptance and Commitment Scale-2 is a widely used measure of psychological flexibility (Bond et al., 2011). It consists of seven items that appraise openness to private thoughts, feelings, sensations, values, and goals without significant defenses. Responses are made on a seven-point continuum from 1 (*Never True*) to 7 (*Always True)*. High scores indicate the absence of psychological flexibility; scores greater than 25 are considered indicators of significant psychological distress, including a greater risk of anxious and depressed symptoms (Berta-Otero et al., 2022; Bond et al., 2011). Alphas ranged from 0.78 to 0.88, and test–retest reliability from 0.79 to 0.81 (Bond et al., 2011). In the present study, alpha was 0.92.

**Multidimensional Scale of Perceived Social Support.** The Multidimensional Scale of Perceived Social Support is a self-report measure with twelve items that measure perceived social support from family, friends, and significant others (Zimet et al., 1988, 1990). Responses are made on a 7-point continuum from 1 (*Strongly Agree*) to 7 (*Strongly Disagree*); lower scores indicate greater perceived social support. Internal consistencies are reported ranging from 0.81 to 0.91 by Zimmet. Alpha in the current study was 0.94.

#### 2.2.3. Criteria

**Impact of Events Scale-Revised.** The Impact of Events Scale-Revised is a twenty-two-item scale developed by Weiss and Marmar (1997). Based on the DSM-IV-TR (American Psychiatric Association, 2000), it includes eight items each to assess intrusion and avoidance, and seven to assess hyperarousal in response to exposure to disasters (Creamer et al., 2003). Responses are made on a five-point continuum from 0 (*Not at All*) to 4 (*Extremely*). Weiss and Marmar (1997) reported an internal consistency coefficient alpha of 0.96; in the present study, alpha was also 0.96.

**International Trauma Scale (ITQ).** The ITQ is a measure of trauma developed by Cloitre et al. (2018). It consists of two subscales of nine items each, intended to measure simple and complex PTSD; the first assesses symptoms of avoidance and sense of threat, while the latter assesses affective dysregulation, negative self-concept, and relationship disruption. Here, we will refer to them as ITQ-A and ITQ-B, respectively. Responses are made on a five-point continuum from 0 (*Not at all)* to 4 (*Extremely*). Camden et al. (2023) reported an alpha of 0.92. Coefficient alphas were 0.91 for ITQ-A and 0.93 for ITQ-B in the present study.

**Satisfaction with Life Scale.** The Satisfaction with Life Scale is a five-item measure to assess self-reported life satisfaction (Pavot & Diener, 1993, 2008). Responses are made on a 7-point continuum from 1 (*Strongly Disagree*) to 7 (*Strongly Agree*); higher scores indicate greater life satisfaction. Validity support has been good. Pavot and Diener reported an internal consistency coefficient alpha of 0.87. In the present study, alpha was 0.91.

### 2.3. Procedures

IRB approval was secured from the George Fox University Human Subjects Research Committee (22030100). Participants were invited to participate in the study through Mechanical Turk. Information about the study was presented on the first page, including the limitation that participants must be eighteen or more years in age. Clicking agreement to participate at the end of the opening page was treated as consent and opened the survey. Respondents first completed the demographics questionnaire. They then completed the eight study measures in a randomly presented order. An Amazon gift card for $10.00 was delivered to their IP address upon completion. Data were gathered in 2021–2022 during the COVID-19 quarantine period.

## 3. Results

Results were examined by means of descriptive statistics, correlations, analyses of variance, and a series of hierarchical regressions in which demographic variables were entered in Model 1, moderator variables of psychological flexibility and perceived social support were entered in Model 2, and disaster experiences were entered in Model 3. In the four regressions examined, the relationship of ND to subjective distress (Impact of Events Scale-Revised), simple and complex PTSD (ITQ-A and ITQ-B), and satisfaction with life (Satisfaction with Life Scale) was examined. Four additional regressions examined the relationship of HDs (ACE and Cumulative Trauma Scale-Revised) with the same four criterion measures.

### 3.1. Natural Disaster

#### 3.1.1. NDS Descriptive Results

The mean NDS score was 1.97 (SD = 2.38), with a median and mode of 1.00. In total, seventy (53.4%) participants reported zero or one ND experience, 43 (32.8%) reported two or three NDS experiences, thirteen (10.0%) reported four to six NDS experiences, and five (3.9%) reported eleven to thirteen NDS experiences. Thus, most participants reported either none or only a single natural disaster experience, but about one-third reported two or three natural disaster experiences, and fourteen percent reported four or more such experiences. See Supplemental Table S1 for the frequency and percent of participants reporting each ND experience.

Descriptive results for the NDS and other study measures are reported in Table 1. Effects of natural and human disasters are reported in the following sections and Supplementary Tables S3–S10. Coefficient alphas ranged from 0.83 to 0.94 for the study scales. Internal consistency coefficient alpha for the NDS was 0.83, indicating good internal consistency.

#### 3.1.2. Factor Analysis

Exploratory factor analysis found that the first factor had an eigenvalue of 6.67 and accounted for 51.3% of the variance. The second factor extracted had an eigenvalue of 1.27 and accounted for 9.8% of the variance. The third factor had an eigenvalue of 1.07 and accounted for 8.2% of the variance. No other factors were suggested.

Forced 1, 2, and 3-factor solutions were examined. Eleven items loaded above 0.42 on a single factor. A forced two-factor solution showed that six items loaded on Factor one, four on Factor 2, two items did not load on either factor, and one item strongly cross-loaded on both factors. The two factors correlated 0.446.

A forced three-factor solution showed that all thirteen items loaded on three factors. Factor 1 included six items: avalanche, bridge/overpass/tunnel collapse, landslide, sinkhole, tsunami, and volcano. Factor 2 included three items: fire, flood, and hurricane/monsoon; Factors 1 and 2 correlated 0.475, so they were strongly related. Factor 3 also included three items: earthquake, which loaded positively, and tornado and blizzard, which correlated negatively. Again, the rockslide item loaded strongly on both Factor 1 and Factor 2. Factor analysis results are reported in Table 2.

Taken together, the factor results of NDs comprise three patterns of exposure to NDs. First, over half the variance accounted for is attributed to Factor 1, a general factor that loaded seven items. The first factor correlated 0.475 with Factor 2, suggesting that the three Factor 2 items of fire, flood, and hurricane/monsoon were closely intertwined, but also strongly related to NDs in Factor 1, possibly due to regional factors, and experiences of rockslides loaded strongly on both these factors. Finally, earthquake exposure and exposure to tornadoes and blizzards were inversely related, suggesting a possible regional pattern where exposure to earthquakes may occur in regions of the US where exposure to tornadoes and blizzards is unlikely to occur, and vice versa.

#### 3.1.3. Item Responses on the NDS

An examination of responses to individual NDS items showed wide disparity in the percentage of participants who reported experiencing specific natural disasters. Over forty percent reported they had experienced a blizzard. More than thirty percent reported they had experienced an earthquake. Over twenty percent reported experiencing a fire, flood, hurricane or monsoon, or tornado. Finally, fewer than ten percent reported experiencing an avalanche or the collapse of a bridge, overpass, or tunnel, landslide, rockslide, sinkhole, tsunami, or volcano. See Supplemental Table S1.

#### 3.1.4. Correlational Results

Pearson’s correlations showed that scores on the NDS are negatively correlated with scores on the Multidimensional Scale of Perceived Social Support, positively correlated with scores on the ACE, Cumulative Trauma Scale-Revised, and Satisfaction with Life Scale, but not correlated with scores on the Acceptance and Commitment Scale-2, Impact of Events Scale-Revised, ITQ-A, or ITQ-B in the present sample. Scores on the Impact of Events Scale-Revised, ITQ-A, and ITQ-B are strongly correlated with each other and correlated inversely but weakly with the Satisfaction with Life Scale. Only the ITQ-B correlated inversely and strongly with the Satisfaction with Life Scale. See Table 3.

Surprisingly, the MSPSS was significantly negatively related to NDS scores; individuals exposed to multiple natural disasters reported greater perceived social support than other participants. The Multidimensional Scale of Perceived Social Support was positively correlated with both the ACE and the Cumulative Trauma Scale-Revised with large effects.

#### 3.1.5. Regression Results

Four hierarchical regressions were computed to appraise the impact of natural disaster on general distress, simple and complex PTSD, and life satisfaction. Standardized scores were used for all regression analyses.

**Impact of Events Scale-Revised.** Hierarchical regressions for the Impact of Events Scale-Revised showed that the demographic items were unrelated to scores on the Impact of Events Scale-Revised in Model 1. The Multidimensional Scale of Perceived Social Support was also unrelated to scores on the Impact of Events Scale-Revised; however, Acceptance and Commitment Scale-2 scores were strongly related with a very large effect (*t* = −8.91; *p* < 0.001; ß = −0.67). NDS scores contributed significant incremental variance in predicting Impact of Events Scale-Revised scores with a small effect (*t* = 2.37; *p* = 0.020; ß = 0.17). See Supplementary Table S3.

**ITQ-A.** The second regression examined the relationship of NDS scores to the ITQ-A. Results showed that the demographic responses were unrelated to scores on the ITQ-A in Model 1. Multidimensional Scale of Perceived Social Support scores were not predictors, but scores on the Acceptance and Commitment Scale-2 were significantly related to scores on the ITQ-A with a large effect (*t* = −8.54, *p* < 0.001, ß = 0.63). NDS scores added significant predictive validity with a small effect (*t* = 2.33, *p* = 0.021, ß = 0.19). See Table 3. See Supplementary Table S4.

**ITQ-B.** The third regression examined the relationship of NDS scores to the ITQ-B scores. Only scores on the Acceptance and Commitment Scale-2 were significantly related to scores on the ITQ-B; they showed a large effect (*t* = 15.35, *p* < 0.001, ß = −0.81). See Supplementary Table S5.

**Satisfaction with Life Scale.** The fourth regression examined the relationship of the NDS to Satisfaction with Life Scale scores. Again, demographic variables were not significant predictors of Satisfaction with Life Scale in Model 1. In Model 2, the Multidimensional Scale of Perceived Social Support was again not a significant predictor; however, Acceptance and Commitment Scale-2 was a significant moderator (*t* = −7.78, *p* < 0.001, ß = −0.56) of Satisfaction with Life Scale with a moderate effect and both age (*t* = −3.83, *p* < 0.001) and education (*t* = 2.32, *p* < 0.001) became significant predictors.

In Model 3, NDS added significant incremental variance that predicted Satisfaction with Life Scale with a small effect (*t* = 4.34, *p* < 0.001, ß = −0.30). Age remained a significant predictor in Model 3 (ß = −0.25) with a small effect, and the Multidimensional Scale of Perceived Social Support also added significant variance in Model 3 (ß = 0.21) with a small effect. See Supplementary Table S6.

### 3.2. Human Disaster

#### Descriptive and Correlational Results

Descriptive and correlational data for the ACE and Cumulative Trauma Scale-Revised are provided in Table 1 and Table 2, respectively. The mean score for ACE was 2.27 (2.65); 94 participants (71.8%) reported fewer than four ACEs; the remaining 37 (28.2%) reported four to ten ACEs. The mean score for the Cumulative Trauma Scale-Revised was 23.89 (4.11).

Correlational findings showed that the ACE and Cumulative Trauma Scale-Revised were strongly related to each other (*r* = 0.628 **), positively related to the Impact of Events Scale-Revised, ITQ-A, and ITQ-B, but unrelated to the Satisfaction with Life Scale.

### 3.3. Regression Summary

**Figure 1:**
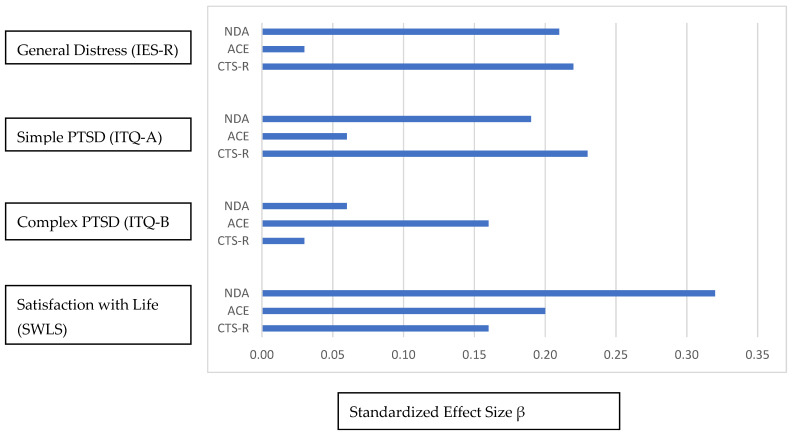
Impact of natural and human disasters on general distress, simple and complex PTSD, and Satisfaction with Life after controlling for demographics, social support, and psychological flexibility.

Figure 1 summarizes regression results. It shows the effect size of ND and HD predictions of simple and complex PTSD, general distress, and life satisfaction. Simple PTSD was predicted by ND and the Cumulative Trauma Scale-Revised. Complex PTSD was predicted by ACE, general distress was predicted by ND and the Cumulative Trauma Scale-Revised, but not by ACE. ND, ACE, and the Cumulative Trauma Scale-Revised all predicted satisfaction with life, but satisfaction with life was more strongly predicted by ND histories.

### 3.4. Supplementary Analyses: Clustering NDS Item Responses

Studies of ACE results have found distinct groupings among participants based on ACE item response patterns (Carlson, 2024; Lacey et al., 2020; Qu, 2022). Thus, a K-cluster analysis using NDS scores was performed for the present sample.

Successful cluster results were created for two, three, and four cluster models. Models with five and six clusters ended up with clusters of a single participant. Here, we examined outcomes associated with the four-cluster model. All participants were classified into four groups of 5, 14, 17, and 95 participants in six iterations.

These four NDS groups differed at *p* < 0.001 on eleven of the thirteen NDS items but did not differ in reports of experiencing a tsunami or a volcano—both of which were rarely reported (=3.8% for each). *F* values ranged from 8.10 for the experience of an earthquake to 215.18 for the experience of a sinkhole. Significantly different patterns of natural disasters (<0.001) were expected as NDS responses were the basis for this grouping.

NDS cluster-groups also differed significantly on ACE (*p* < 0.001), Multidimensional Scale of Perceived Social Support (*p* = 0.023), Cumulative Trauma Scale-Revised (*p* < 0.001), and Satisfaction with Life Scale (*p* = 0.050) scores. But NDS cluster-based groups did not differ on the AAQ-II, ITQ-A, or ITQ-B. See Supplemental Table S11.

**Group 1: Every NDS.** Group 1 was the smallest group; it consisted of five participants (3.9%); four reported they had experienced all thirteen forms of ND; one reported they had experienced all NDs but a flood and a landslide. This group also reported the most ACEs (M = 9.4; SD = 1.34) and the highest Cumulative Trauma Scale-Revised scores. Four reported all 10 ACE items, while one reported 7. On the Cumulative Trauma Scale-Revised, four reported Yes to all twenty items, while the remaining individual reported Yes on eighteen items. Thus, they reported that they were multiply unfortunate due to both natural and human disasters. They also reported significantly higher Cumulative Trauma Scale-Revised and lower Multidimensional Scale of Perceived Social Support scores than the other participants, yet their SWLS scores were higher than all but one of the other NDS groups. Their scores did not differ from the other NDS groups on the Acceptance and Commitment Scale-2, Impact of Events Scale-Revised, ITQ-A, and ITQ-B.

**Group 2: Low NDS.** Most participants were among the 95 participants (72.5%) in Group 2 who reported the lowest scores on the NDS (0.89/0.78). Most had experienced at most one natural disaster. Thirty-three (24.7%) reported they had experienced a blizzard, twenty-seven (28.4%) an earthquake, eleven (11.6%) a tornado, and ten (10.5%) reported undergoing a fire or hurricane/monsoon. This group also reported the lowest ACE (1.89/2.22) and Cumulative Trauma Scale-Revised (2.98/2.70) scores.

**Group 3: Flood.** Group 3 included seventeen participants (2.82/1.42). Among these, NDS scores were moderate, but fourteen had experienced a flood, and most had experienced a couple of other natural adversities; over half reported experiencing fire, flood, hurricane/monsoon, or tornado.

**Group 4 Blizzard Plus.** Group 4 consisted of fourteen individuals who reported mostly two to four natural adversities (3.00/1.18). Over seventy percent of this group reported undergoing earthquakes, floods, and blizzards. One-third also reported undergoing fires and hurricanes/and monsoons.

## 4. Discussion

Study goals were two-fold. First, the development of a scale to measure the experience of multiple natural disasters, enabling the study of their aggregate impact rather than limiting the study to only a single disastrous event. Second, shedding light on how a lifetime of natural disasters may affect human functioning in terms of psychological distress and comparing the effects of accumulated natural disasters with the better-known effects of human adversity/disaster.

### 4.1. Measuring Natural Disaster Exposure

***Internal Consistency.*** Results for the measurement of NDs are somewhat mixed. The scale proved to have adequate internal consistency, a result suggesting multiple NDs often occur to individuals—or perhaps more accurately, retrospective reports of having undergone natural trauma occur together. This finding is parallel to those for adverse childhood experiences. The ACE was originally conceived of as a simple checklist; however, subsequent studies have shown strong internal consistency coefficients for the ACE (e.g., Bufford et al., 2017) and that individual adverse experiences tend to occur in clusters (Carlson, 2024; Lacey et al., 2020; Qu, 2022).

### 4.2. NDS Validation

***Factor Analyses.*** Exploratory factor analysis was performed. A scree plot suggested one to three factors. The first factor had an eigenvalue of 6.67 and accounted for 51.3% of the variance. The second factor had an eigenvalue of 1.27 and accounted for 9.8% of the variance. The third factor had an eigenvalue of 1.07 and accounted for 8.2% of the variance.

Forced 1, 2, and 3-factor solutions were examined. Eleven items loaded above 0.42 on a forced single factor; alpha for this 11-item scale was 0.85. Only the experiences of a blizzard or an earthquake were excluded. These items are loaded in opposite directions on Factor 3 in the three-factor solutions. Earthquakes and blizzards tend to occur in different regions of the United States, suggesting that exposure to one of these NDs was associated with reduced likelihood of exposure to the other.

Examination of a forced two-factor solution showed that seven items loaded on Factor 1, four on Factor 2, and two items did not load cleanly on either factor. The experience of a rockslide strongly loaded on both factors (0.777 and 0.640), while the experience of a tornado cross-loaded on Factor 1 and loaded on Factor 2 (0.379, 0.524). The two factors correlated 0.446. Alphas were 0.96 and 0.52 for Factors 1 and 2, respectively.

A forced three-factor solution showed that twelve items loaded on three factors; the thirteenth item, rockslide, loaded strongly on both Factor 1 and Factor 2 (0.796, 0.642). Factor 1 included six items: avalanche, bridge/overpass/tunnel collapse, landslide, sinkhole, tsunami, and volcano. Factor 2 included three items: fire, flood, and hurricane/monsoon; Factors 1 and 2 correlated 0.475, so they were strongly related; Factor 2 was identical for both the two-factor and three-factor solutions, and their correlations were quite similar. Factor 3 also included three items: earthquake, which loaded positively; tornado and blizzard loaded negatively. Alphas were 0.96, 0.57, and 0.16 for Factors 1, 2, and 3, respectively. Thus, the three-factor solution was also deemed unsatisfactory. The one-factor solution was deemed most satisfactory.

***Correlational Findings.*** Correlational data showed that NDS scores were positively related to social support, general distress, and satisfaction with life, albeit with small effects. NDS scores were unrelated to simple and complex PTSD symptoms.

The correlations with HD were surprisingly strong. On the face of it, natural and HD seem to be independent events. So, what gives? It appears that NDs and HDs come together in some way. Perhaps our memories are biased. Or maybe undergoing one form of disaster makes us more sensitive to the other. A third possibility is that natural and human disasters become intertwined; for example, returning home from evacuation for Hurricane Katrina and finding my home was vandalized or my possessions stolen in my absence.

Seeking social support and human aid in the aftermath of natural disasters is likely a normal response. Sadly, at such times, fellow humans may simply fail us; worse, they may sometimes exploit us. Thus, ND and HD may become intertwined, and in the end, the HD may weigh more heavily on us.

A second somewhat unexpected discovery was that NDS scores were somewhat positively correlated with perceived social support, suggesting that those who undergo natural disasters may receive above-average social support. In contrast, perceived social support is negatively related to human disaster. This contrasting effect on access to social support may account for a significant part of the adverse effects of human disaster, especially with respect to complex PTSD. HD may impair trust, reduce support-seeking behavior, and stem from or contribute to alienation from others. Some data suggest that alienation from self may also be a result of HD (Bufford et al., 2017). Together or separately, these processes could readily impair the reception of social support following HD.

### 4.3. Natural and Human Disaster

The second goal of the study was to compare the levels of distress/trauma that follow ND and HD. In this study, hierarchical regression was used to control demographic factors, psychological flexibility, and social support to refine the appraisal of the relationship between disaster experiences and psychological distress/trauma, simple and complex PTSD, and life satisfaction. Demographic factors were not significantly related to any criterion except that age was a negative predictor of life satisfaction with a small effect; older participants reported slightly lower life satisfaction (*r* = −0.18). Of the two moderator variables, psychological flexibility proved a consistent predictor, while social support only predicted life satisfaction.

***Natural Disaster.*** Psychological flexibility was a significant moderator with a moderate effect on ND experiences. It was related to lower levels of psychological distress, simple PTSD, and complex PTSD; it was also associated with greater life satisfaction. However, social support had little discernible relationship to these outcomes. ND had a small incremental effect on psychological distress, simple PTSD, and life satisfaction, but was not incrementally related to complex PTSD.

NDs were found to show four different patterns among participants. These included a majority of participants with an average of less than one natural disaster experience. Two small groups of participants averaged about three natural disasters; the first group commonly experienced flooding, while the second commonly experienced blizzards. Finally, a fourth group of five participants reported they had experienced all or nearly all thirteen NDs.

Distinctions among groups two and three could be related to geographical factors, as avalanches, hurricanes, and tornadoes generally occur in specific geographic regions. But people move over a lifetime, so they may experience multiple regional patterns. Still, it was surprising that five (3.8%) reported experiencing eleven or more individual NDs. There may be an untold story here. Reports of undergoing all forms of natural disasters could be accurate. But they could be a result of a bias for saying Yes, inattentive responses, or robotic responses. Inaccurate memory might also be a factor. As no strategies were employed to identify invalid responses in conducting this survey, robotic and inattentive responses cannot be ruled out.

***Human Disaster.*** As with NDs, demographic characteristics were included in Model 1 for all analyses but did not predict general distress, simple or complex PTSD, or satisfaction with life, except that age was a negative predictor of life satisfaction with a small effect; older participants reported lower life satisfaction.

Psychological flexibility showed significant protective effects for all regressions that examined the effect of human disaster. Surprisingly, while adverse childhood experiences incrementally increased general distress, complex PTSD, and satisfaction with life, adult HDs had a significant small effect only in the prediction of simple PTSD, for which childhood adversity was not a significant predictor. The often-later timing of adult HD may affect its trajectory in terms of traumatic impact.

Social support did not affect general distress or complex PTSD. But it had a small effect on simple PTSD and life satisfaction in these analyses.

Taken together, these regressions show generally similar effects of ND and HD in predicting symptoms of general psychological distress, simple PTSD, and impaired satisfaction with life. For complex PTSD, only HD in the form of adverse childhood experiences proved to be a significant predictor.

So, what can we conclude? HD and ND both have significant adverse relationships with psychological functioning. Which has a stronger impact remains uncertain, though the present findings lean a bit toward human disaster.

Psychological flexibility consistently predicted lesser distress following both human and natural disasters. Surprisingly, in these participants, social support was largely unrelated to their distress, except for predicting greater satisfaction with life and some small moderating effect on the effects of adult HD on simple PTSD.

Limitations include sample characteristics. While a nationally based US sample, the sample remains a convenient sample of persons who engage in taking surveys for compensation. Their education and technical savvy are likely above average. The Impact of Events Scale-Revised was developed based on an earlier DSM that did not include negative alterations of mood and cognition added to the definition of PTSD in DSM-5 (American Psychiatric Association, 2013). Cultural and geographic features also could alter the relationships reported here, especially outside the US.

Refinements to the NDS may be warranted. But the NDS provides a suitable measure of the experience of natural disasters and can enable further exploration of how undergoing natural disasters shapes individual functioning, including fostering traumatic or resourceful outcomes.

### 4.4. Practical Implications

What might these findings mean for practice? Attention to the patient’s current levels of distrust in him/herself, in others, in the world, in the future (or hopefulness), and in God—or R/S struggle (Hansen et al., 2023; Pargament & Exline, 2022; Wilt et al., 2019, 2022) seem to be critical areas for intervention. Due to the important role of psychological flexibility in ameliorating the effects of NDs, ACT interventions or similar approaches within other therapeutic models are recommended.

Central to a therapy relationship is gaining trust in the therapist and hope for the future. These will be significantly affected by the degree to which trust has been compromised. Developing a working therapeutic alliance and a sense of hope for the future seem to be essential initial therapeutic challenges. In turn, subsequent areas for treatment include fostering the development of the capacity to test trustworthiness in ways that minimize risk and promote practical wisdom. Fostering wisely tested trust is important in other human relationships, in life circumstances, and in R/S struggles (Kira & Ferreira, 2022). Finally, promoting psychological flexibility, the capacity to engage non-defensively with the full range of life experiences—to foster resourcefulness and resilience seems another important clinical emphasis (Chisari et al., 2022; Dixon et al., 2023; Hayes et al., 2017).

Finally, the strength of the relationship between childhood adversity and complex PTSD is similar in magnitude to the other adversity and outcome relationships. But treatment for complex PTSD seems to pose greater challenges. In turn, this suggests that in some ways not identified here, the impact of childhood adversity may be greater than that of natural disasters and more general (or developmentally later) human disasters.

### 4.5. Recommendations for Future Research

As suggested by a reviewer, robotic and inattentive responses have become a plaguing problem with internet surveys. Items to identify and remove such responses should be included in any future online ND research. Inclusion of individuals across cultural, regional, and national boundaries is also desired, as the present results suggest that where one lives may influence ND exposure, and individual and cultural differences may influence responses to such exposure.

### 4.6. Summary and Conclusions

In summary, the Natural Disaster Scale passed initial tests of reliability and validity. Results showed that those with more exposure to natural disasters tended to report lower general well-being, more symptoms of simple PTSD, and lower life satisfaction. But exposure to natural disasters did not contribute to predicting complex PTSD. Human disasters had a similar magnitude of effects on the criterion measures, but adult adversity/disaster predicted general distress, simple PTSD, and life satisfaction, while childhood human adversity predicted complex PTSD. For this sample, demographic characteristics had little effect on outcome measures aside from the finding that older participants had slightly lower satisfaction with life and education was associated with a small increase in life satisfaction. These data point toward generally similar magnitudes of outcomes for natural and human disasters. Finally, promoting psychological flexibility in ways such as those proposed in ACT treatment is suggested as a promising intervention.

## Figures and Tables

**Table 1:** Coefficient alphas and descriptive results for study measures.

Scale	Alpha	Mean	SD	Skew	Kurtosis
***Predictors***					
ACE	0.84	1.97	2.38	1.27	0.97
CTS-R	0.89	23.89	4.11	2.42	6.61
NDS	0.83	1.97	2.38	2.80	9.08
***Moderators***					
AAQ-2	0.92	20.76	9.85	0.34	−0.92
MSPSS	0.94	65.92	15.42	−1.33	1.68
***Criteria***					
IES-R	0.96	46.85	21.33	0.70	−0.23
ITQ-A	0.91	17.88	8.47	0.93	0.08
ITQ-B	0.93	18.20	8.71	0.91	0.19
SWLS	0.91	15.83	7.20	0.74	−0.04

Note: N = 131; SE skew = 0.212 and SE Kurtosis = 0.420 for all measures; ACE = Adverse Childhood Experiences scale, CTS-R = Cumulative Trauma Scale-Revised, NDS = Natural Disaster Scale, AAQ-2 = Acceptance and Commitment Scale, 2nd edition, MSPSS = Multidimensional Scale of Perceived Social Support, IES-R = Impact of Events Scale-Revised, ITQ-A = International Trauma Questionnaire-PTSD, ITQ-B = International Trauma Questionnaire-Complex PTSD. SWLS = Satisfaction with Life Scale.

**Table 2:** Factor loadings for the natural disaster scale one-factor structure matrix.

Item	Factor 1
NDS-1 Avalanche	**0.907**
NDS-2 Bridge/overpass/tunnel collapse	**0.869**
NDS-3 Earthquake	0.340
NDS-4 Fire	**0.493**
NDS-5 Flood	**0.421**
NDS-6 Hurricane/Monsoon	**0.440**
NDS-7 Landslide	**0.838**
NDS-8 Rockslide	**0.835**
NDS-9 sinkhole	**0.924**
NDS-10 Tornado	**0.469**
NDS-11 Tsunami	**0.953**
NDS-12 Volcano	**0.953**
NDS-13 Blizzard	0.275

Note: N = 131. Alpha = 0.85.

**Table 3:** Alpha coefficients and correlations among study scales.

Scales	Alpha	ACE	CTS-R	NDS	AAQ-2	MSPSS	IES-R	ITQ-A	ITQ-B
**Predictors**									
ACE	0.84								
CTS-R	0.89	0.628 **							
NDS	0.85	0.494 **	0.709 **						
**Moderators**									
AAQ-2 ^@^	0.92	−0.344 **	−0.213 *	−0.023					
MSPSS	0.94	−0.444 **	−0.316 **	−0.275 **	0.262 **				
**Criteria**									
IES-R	0.96	0.335 **	0.313 *	−0.157	−0.643 **	−0.106			
ITQ-A	0.91	0.305 **	0.317 **	0.108	−0.595 **	−0.077	0.716 **		
ITQ-B	0.93	0.427 **	0.292 **	0.093	−0.811 **	−0.299 **	0.646 **	0.672 **	
SWLS	0.91	−0.047	0.068	0.245 **	0.573 **	0.305 **	−0.206 *	−0.229 **	−0.510 **

**Note**: N = 131; * *p* < 0.05, ** *p* < 0.01; ACE = Adverse Childhood Experiences scale, CTS-R = Cumulative Trauma Scale-Revised, NDS = Natural Disaster Scale, AAQ-2 = Acceptance and Commitment Scale, 2nd edition—(reverse-scored), MSPSS = Multidimensional Scale of Perceived Social Support, IES-R = Impact of Events Scale-Revised, ITQ-A = International Trauma Questionnaire-PTSD, ITQ-B = International Trauma Questionnaire-Complex PTSD. SWLS = Satisfaction with Life Scale. ^@^ The AAQ-2 is reverse-scored; high scores signify the absence of psychological flexibility.

## Data Availability

Data are available by request to the senior author.

## Supplementary Materials

The following supporting information can be downloaded at https://www.mdpi.com/article/10.3390/bs15070848/s1. Table S1: Item Responses to Natural Trauma Scale Items; Table S2: Hierarchical Regression of Incremental Validity of NDS-11 in Predicting IES-R after controlling for Demographic Variables and Moderator Variables of AAQ-2, and MSPSS; Table S3: Hierarchical Regression of Incremental Validity of NDS-11 in Predicting ITQ-A after controlling for Demographic Variables and Moderator Variables of AAQ-2 and MSPSS; Table S4: Hierarchical Regression of Incremental Validity of NDS-11 in Predicting ITQ-B after controlling for Demographic Variables and Moderator Variables of AAQ-2 and MSPSS; Table S5: Hierarchical Regression of Incremental Validity of NDS in Predicting SWLS after Controlling for Demographic Variables and Moderator Variables of AAQ-2 and MSPSS; Table S6: Hierarchical Regression of Incremental Validity of ACE and CTS-R in Predicting IES-R after controlling for Demographic Variables and Moderator Variables AAQ-2 and MSPSS; Table S7: Hierarchical Regression of Incremental Validity of ACE and CTS-R in Predicting ITQ-A after controlling for Demographic Variables and Moderator Variables AAQ-2 and MSPSS; Table S8: Hierarchical Regression of Incremental Validity of ACE and CTS-R in Predicting after controlling for Demographic Variables and Moderator Variables AAQ-2 and MSPSS; Table S9: Hierarchical Regression of Incremental Validity of ACE and CTS-R in Predicting SWLS after controlling for Demographic Variables and Moderator Variables AAQ-2 and MSPSS; Table S10: Analyses of Variance Comparing NDS Cluster-Based Groups on Study Scales; Table S11: Natural and Human Disaster: Comparing Effects.
