# Supplementary material for: Natural Disasters, Psychosocial Distress, Psychological Flexibility, and Satisfaction with Life
Source: Behav Sci (Basel). 2025 Jun 24;15(7):848. doi: 10.3390/bs15070848 (PMC12292627; doi:10.3390/bs15070848)
Supplement: Supplementary file 1 [file behavsci-15-00848-s001.zip › behavsci-3518306-supplementary.pdf]

Supplemental Table S1

Item Responses to Natural Trauma Scale Items

Item	YES #	%	NO #	%
1. Avalanche	6	4.6	125	95.4
2. Blizzard	55	42.7	76	57.3
3. Bridge, tunnel, overpass collapse	7	5.3	124	94.7
4. Earthquake	45	34.4	86	65.6
5. Fire	31	23.7	100	76.3
6. Flood	31	23.7	100	76.3
7. Hurricane or monsoon	31	23.7	100	76.3
8. Landslide	5	4.6	126	95.4
9. Rockslide	9	6.9	122	93.1
10. Sinkhole	6	4.6	125	95.4
11. Tornado	27	20.6	104	79.4
12. Tsunami	5	3.8	126	96.2
13. Volcano	5	3.8	126	96.2

Note: N = 131

Supplemental Table S2

Hierarchical Regression of Incremental Validity of NDS-11 in Predicting IES-R after controlling for Demographic Variables and Moderator Variables of AAQ-2, and MSPSS

Model	Added Predictors	R	R^2	ΔR^2	*F*	df	Sig	B [CI]	ß	*t*	Sig	*r*	r_p
Model 1													
	Demographics	.250	.062	.062	1.64	5,123	.156						
Model 2	AAQ-2 & MSPSS	.661	.437	.375	13.42	7,121	<.001						
	AAQ-2							-1.34 [-1.64/-1.04]	-0.67	-8.91	<.001	-.64	-.63
	MSPSS							0.10 [-0.09/0.29]	0.07	1.03	.30	.14	.14
Model 3		.680	.462	.025	12.89	8,120	<.001						
	AAQ-2							-1.37 [-1.66/-1.07]	-0.65	-9.23	<.001	-.64	-.64
	MSPSS							0.19 [-0.01/0.39]	0.13	1.72	.089	.14	.17
	NDS-11							1.76 [0.29/3.23s]	0.17	2.37	.020	.16	.21

Note: *N*= 131. Insignificant demographic predictors are omitted. AAQ-2 = Acceptance and Commitment Scale-2, MSPSS = Multidimensional Scale of Perceived Social Support, NDS = Natural Disaster Scale. IES-R = Impact of Events Scale-Revised. All scales were normalized for regression analyses.

Supplemental Table S3

Hierarchical Regression of Incremental Validity of NDS-11 in Predicting ITQ-A after controlling for Demographic Variables and Moderator Variables of AAQ-2 and MSPSS

Model	Added Predictors	R	R^2	ΔR^2	*F*	df	Sig	B [cl]	ß	*t*	Sig	*r*	r_p
Model 1	Demographics	.070	.005	.005	0.12	5,123	.987						
Model 2	AAQ-2 & MSPSS	.627	.365	.360	9.96	7,121	<.001						
	AAQ-2							-0.48 [0.60/0.37]	-0.63	-8.24	<.001	-.60	-.60
	MSPSS							0.40 [-0.36/0.12]	0.08	1.04	.299	-.08	.09
Model 3		.627	.393	.027	9.71	8,120	<.001						
	AAQ-2							-0.49 [-0.61/-0.38]	0.63	-8.54	<.001	-.62	-.61
	MSPSS							0.07 [-0.01/0.14]	0.14	1.71	.089	.15	.12
	NDS							0.67 [0.10/1.24]	0.19	2.33	.021	.14	.21

Note: *N*= 131. Insignificant demographic predictors are omitted. AAQ-2 = Acceptance and Commitment Scale-2, MSPSS = Multidimensional Scale of Perceived Social Support, NDS Natural Disaster Scale. ITQ-A = International Trauma Scale-Simple PTSD. All scales were normalized for regression analyses.

Supplemental Table S4

Hierarchical Regression of Incremental Validity of NDS-11 in Predicting ITQ-B after controlling for Demographic Variables and Moderator Variables of AAQ-2 and MSPSS

**Model**	**Added Predictors**	**R**	**R^2**	**ΔR^2**	***F***	**df**	**Sig**	**B [cl]**	**ß**	***t***	**Sig**	***r***	**r_p**
Model 1	Demographics	.188	.035	.035	0.90	5,123	.483						
Model 2	AAQ-2 & MSPSS	.833	.694	.659	39.21	7,121	<.001						
	AAQ-2							-0.72 [-0.81/-0.63]	-0.81	15.35	<.001	-.82	-.81
	MSPSS							-0.04 [-0.10/0.02]	-0.07	-1.27	.205	-.28	-.12
Model 3		.835	.698	.004	34.61	8,120	<.001						
	AAQ-2							-0.72 [-0.82/-0.63]	-0.81	15.42	<.001	-.82	-.78
	MSPSS							-0.03 [-0.09/0.04]	-0.05	-0.86	.393	-.08	-.04
	NDS							0.28 [-0.17/0.75]	0.06	1.19	.237	.11	.06

Note: *N*= 131. Insignificant demographic predictors are omitted. AAQ-2 = Acceptance and Commitment Scale-2, MSPSS = Multidimensional Scale of Perceived Social Support, NDS Natural Disaster Scale. ITQ-B = International Trauma Scale-Complex PTSD. All scales were normalized for regression analyses.

Supplemental Table S5

Hierarchical Regression of Incremental Validity of NDS in Predicting SWLS after Controlling for Demographic Variables and Moderator Variables of AAQ-2 and MSPSS

Model	Added Predictors	R	R^2	ΔR^2	*F*	df	Sig	B [cl]	ß	*t*	Sig	*r*	r_p
Model 1	Demographics	.284	.081	.081	2.16	5,123	.063						
Model 2	AAQ-2 & MSPSS	.654	.428	.347	12.91	7,121	<.001						
	Age							-1.63 [-2.45/-0.79]	-0.29	-3.83	<.001	-.20	-.33
	Education							0.69 [0.10/1.27]	-0.25	0.17	2.32	.04	.21
	AAQ-2							0.41 [0.30/0.51]	0.56	-7.78	<.001	.57	.58
	MSPSS							0.06 [-0.01/0.12]	0.12	1.67	.098	.27	.15
Model 3		.711	.513	.078	15.32	8,120	<.001						
	Age							-1.41 [-2.20/-0.62]	- 0.25	-3.53	< .001	-.20	-.31
	AAQ-2							0.39 [0.29/0.49]	0.54	8.00	<.001	.57	.59
	MSPSS							0.10 [0.03/0.17]	0.21	3.01	.003	.27	.27
	NDS							1.06 [0.58/1.54]	0.30	4.34	<.001	.25	.37

Note: *N*= 131. Insignificant demographic predictors are omitted. AAQ-2 = Acceptance and Commitment Scale-2, MSPSS = Multidimensional Scale of Perceived Social Support, NDS Natural Disaster Scale. SWLS = Satisfaction with Life Scale. All scales were normalized for regression analyses.

Supplemental Table S6

Hierarchical Regression of Incremental Validity of ACE and CTS-R in Predicting IES-R after controlling for Demographic Variables and Moderator Variables AAQ-2 and MSPSS

Model	Added Predictors	R	R^2	ΔR^2	*F*	df	Sig	B [cl]	ß	*t*	Sig	*r*	r_p
Model 1	Demographics	.252	.063	.063	1.66	5,123	.148						
Model 2	AAQ-2 & MSPSS	.664	.441	.378	13.66	7,121	<.001						
	AAQ-2							0.65 [0.50/0.79]	0.64	8.98	<.001	.65	.63
	MSPSS							0.07 [-0.07/0.22]	0.07	1.00	.318	-.11	.09
Model 3		.697	.486	.044	12.49	9,119	<.001						
	AAQ-2							0.61 [0.47/0.75]	0.60	8.51	<.001	.65	.62
	MSPSS							0.15 [< 0.00/0.30]	0.15	1.96	.053	-.11	.18
	ACE							0.03 [-0.16/0.22]	0.03	0.33	.746	.33	.03
	CTS-R							0.22 [0.04/0.39]	0.22	2.41	.018	.32	.22

Note: *N*= 131. Insignificant predictors are omitted. AAQ-2 = Acceptance and Commitment Scale-2, MSPSS = Multidimensional Scale of Perceived Social Support, NDS Natural Disaster Scale. SWLS = Satisfaction with Life Scale. All scales were normalized for regression analyses.

Supplemental Table S7

Hierarchical Regression of Incremental Validity of ACE and CTS-R in Predicting ITQ-A after controlling for Demographic Variables and Moderator Variables AAQ-2 and MSPSS

Model	Added Predictors	R	R^2	ΔR^2	*F*	df	Sig	B [cl]	ß	*t*	Sig	*r*	r_p
Model 1	Demographics	.065	.004	.004	0.11	5,123	.991						
Model 2	AAQ-2 & MSPSS	.607	.368	.364	10.07	7,121	< .001						
	AAQ-2							0.64 [0.48/0.79]	0.63	8.30	<.001	.60	.60
	MSPSS							0.08 [-0.07/0.24]	0.08	1.07	.285	-.07	.10
Model 3		.653	.427	.059	9.85	9,119	< .001						
	AAQ-2							0.59 [0.44/0.74]	0.58	7.80	<.001	.60	.58
	MSPSS							0.18 [0.02/0.34]	0.17	2.17	.032	-.07	.20
	ACE							0.06 [-0.14/0.26]	0.06	0.58	.565	.31	.05
	CTS-R							0.23 [0.05/0.42]	0.23	2.47	.015	.33	.22

Note: *N*= 131. Insignificant predictors are omitted. AAQ-2 = Acceptance and Commitment Scale-2, MSPSS = Multidimensional Scale of Perceived Social Support, NDS Natural Disaster Scale. SWLS = Satisfaction with Life Scale. All scales were normalized for regression analyses.

Supplemental Table S8

Hierarchical Regression of Incremental Validity of ACE and CTS-R in Predicting after controlling for Demographic Variables and Moderator Variables AAQ-2 and MSPSS

Model	Added Predictors	R	R^2	ΔR^2	*F*	df	Sig	B [cl]	ß	*t*	Sig	*r*	r_p
Model 1	Demographics	.188	.035	.035	0.90	5,123	.483						
Model 2	AAQ-2 & MSPSS	.833	.694	.659	39.21	7,121	<.001						
	AAQ-2							0.81 [0.71/0.92]	0.81	15.35	<.001	.82	.81
	MSPSS							-0.07 [-0.18/0.04]	-0.07	-1.27	.205	-.28	-.12
Model 3		.846	.716	.022	33.37	9,119	<.001						
	AAQ-2							0.78 [0.67/0.88]	0.77	14.60	<.001	.82	.80
	MSPSS							< 0.00 [-0.11/0.11]	<-0.01	-0.02	.984	-.28	< -.01
	ACE							0.16 [0.02/0.30]	0.16	2.24	.027	.44	.20
	CTS-R							0.03 [-0.11/0.16]	0.03	0.39	.701	.30	.04

Note: *N*= 131. Insignificant predictors are omitted. AAQ-2 = Acceptance and Commitment Scale-2, MSPSS = Multidimensional Scale of Perceived Social Support, NDS Natural Disaster Scale. SWLS = Satisfaction with Life Scale. All scales were normalized for regression analyses.

Supplemental Table S9

Hierarchical Regression of Incremental Validity of ACE and CTS-R in Predicting SWLS after controlling for Demographic Variables and Moderator Variables AAQ-2 and MSPSS

Model	Added Predictors	R	R^2	ΔR^2	*F*	df	Sig	B [cl]	ß	*t*	Sig	*r*	r_p
Model 1	Demographics	.284	.081	.081	2.16	5,123	.063						
	Age							-0.19 [-0.33/-0.04]	-0.24	-2.55	.012	-.20	-.22
Model 2	AAQ-2 & MSPSS	.654	.428	.347	12.91	7,121	<.001						
	Age							-0.23 [-0.34/-0.11]	-0.29	-3.83	< .001	-.20	-.33
	Education							0.10 [0.01/0.18]	0.17	2.32	.022	.04	.21
	AAQ-2							-0.55 [-0.70/-0.41]	-0.56	-7.78	< .001	-.57	-.58
	MSPSS							0.12 [-0.02/0.26]	0.12	1.67	.098	.27	.15
Model 3		.708	.502	.074	13.32	9,119	<.001						
	Age							-0.23 [-0.34/-0.12]	-0.29	-4.13	< .001	-.20	-.35
	Education							0.07 [0.00/0.15]	0.13	1.89	.061	.04	.17
	AAQ-2							-0.62 [-0.75/-0.48]	-0.62	-8.94	< .001	-.57	-.63
	MSPSS							0.24 [0.09/0.39}	0.24	3.23	.002	.27	.28
	ACE							0.19 [0.01/0.38]	0.20	2.05	.042	-.05	.19
	CTS-R							0.16 [-0.02/0.33]	0.16	1.81	.073	.05	.16

Note: *N*= 131. Insignificant predictors are omitted. AAQ-2 = Acceptance and Commitment Scale-2, MSPSS = Multidimensional Scale of Perceived Social Support, ACE = Adverse Childhood Experiences, SWLS = Satisfaction with Life Scale. All scales were normalized for regression analyses

Supplemental Table S10

Analyses of Variance Comparing NDS Cluster-Based Groups on Study Scales

	N	M	SD	df	F	Sig	Post Hoc Comparison
**ACE**							
Group 1	5	9.40	1.34				
Group 2	95	1.89	2.22				
Group 3	17	2.35	2.42				
Group 4	14	2.14	2.51				
Total	131	2.27	2.65	3, 127	17.51	< .001	Group 1 > Group 2 = 3 = 4
**CTS-R**							
Group 1	5	18.80	2.68				
Group 2	95	2.98	2.70				
Group 3	17	4.18	2.30				
Group 4	14	4.36	3.93				
Total	131	3.89	4.11	3, 127	50.66	< .001	Group 1 > Group 2 = 3 = 4
**NDS**[#]							
Group 1	5	11.60	0.89				
Group 2	95	0.89	0.78				
Group 3	17	2.82	1.42				
Group 4	14	3.00	1.18				
Total	131	1.78	2.33	3, 127	288.07	< .001	Group 1 > Group 2 = 3 = 4
**AAQ-2**[@]							
Group 1	5	32.60	17.73				
Group 2	95	35.05	9.52				
Group 3	17	38.76	9.90				
Group 4	14	33.14	8.55				
Total	131	35.24	9.85	3, 127	1.07	.364	
**MSPSS**							
Group 1	5	41.60	32.57				
Group 2	95	67.38	13.53				
Group 3	17	67.88	13.67				
Group 4	14	62.29	15.35				
Total	131	65.92	15.42	3, 127	5.25	.002	Group 1 < Group 2 = 3 = 4

Supplemental Table S10 continued
Analyses of Variance Comparing NDS Cluster-Based Groups on Study Scales

	N	M	SD	df	F	Sig	Post Hoc Comparison
**IES-R**							
Group 1	5	59.20	38.45				
Group 2	95	43.12	19.42				
Group 3	17	40.53	17.88				
Group 4	14	56.64	18.74				
Total	131	44.84	20.50	3, 127	2.97	.034	Group 4 > Group 2 = 3; Group 1 = 2 = 3
**ITQ-A**							
Group 1	5	21.80	13.97				
Group 2	95	15.69	6.98				
Group 3	17	14.59	8.56				
Group 4	14	15.64	6.59				
Total	131	15.78	7.49	3, 127	1.23	.301	
**ITQ-B**							
Group 1	5	25.00	16.36				
Group 2	95	18.32	8.31				
Group 3	17	16.06	9.65				
Group 4	14	17.57	6.21				
Total	131	18.20	8.71	3, 127	1.40	.246	
**SWLS**							
Group 1	5	32.40	2.61				
Group 2	95	23.29	7.25				
Group 3	17	26.12	6.82				
Group 4	14	24.79	6.42				
Total	131	24.17	7.20	3, 127	3.26	.024	Group 1 > Group 2 = 4 Group 2 = 3 = 4

Note: N = 131; ACE = Adverse Childhood Experiences scale, CTS-R = Cumulative Trauma Scale-Revised, NDS = Natural Disaster Scale, AAQ-2 = Acceptance and Commitment Scale, 2nd edition—(reverse-scored), MSPSS = Multidimensional Scale of Perceived Social Support, IES-R = Impact of Events Scale-Revised, ITQ-A = International Trauma Questionnaire-PTSD, ITQ-B = International Trauma Questionnaire-Complex PTSD. SWLS = Satisfaction with Life Scale.
#Note that clustering was based on NDS, so NDS groups are expected to differ.
@The AAQ-2 is reverse-scored; high scores signify the absence of psychological flexibility.

Supplemental Table S11

Natural and Human Disaster: Comparing Effects

Criterion	Natural Disaster		Human Disaster			
	NDS		ACEs		CTS-R	
	r	*β*[1]	*r*	*β*	*r*	*β*
Moderator						
AAQ-2[@]	.039	-0.65**	-.344**	-0.60**	-.213*	0.60**
MSPSS	-.291**	0.14	-.444**	0.15**	-.316**	0.15
Criteria						
IES-R	.192*	0.21**	.335**	0.03	.313**	0.22*
ITQ-A	.160	0.19**	.305**	0.06	.317**	0.23*
ITQ-B	.108	0.06	.427**	0.16*	.292**	0.03
SWLS	.224*	0.32**	-.047	0.20*	.068	0.16

Note: N = 131; ACE = Adverse Childhood Experiences scale, CTS-R = Cumulative Trauma Scale-Revised, NDS = Natural Disaster Scale, AAQ-2 = Acceptance and Commitment Scale, 2nd edition—(reverse-scored), MSPSS = Multidimensional Scale of Perceived Social Support, IES-R = Impact of Events Scale-Revised, ITQ-A = International Trauma Questionnaire-PTSD, ITQ-B = International Trauma Questionnaire-Complex PTSD. SWLS = Satisfaction with Life Scale.

β = standardized effect size.

[@]The AAQ-2 is reverse-scored; high scores signify the absence of psychological flexibility.
